# Survival with dacarbazine and fotemustine in newly diagnosed glioblastoma multiforme

**DOI:** 10.1038/sj.bjc.6600769

**Published:** 2003-02-18

**Authors:** B Fazeny-Dörner, M Veitl, C Wenzel, K Rössler, K Ungersböck, K Dieckmann, M Piribauer, J Hainfellner, C Marosi

**Affiliations:** 1 Clinical Division of Oncology & Ludwig Boltzmann Institute for Clinical & Experimental Oncology, Department of Internal Medicine I, Währinger Gürtel 18–20, University of Vienna; A-1090 Vienna, Austria; 2 Clinical Institute for Medical and Chemical Laboratory Diagnostics, University of Vienna, A-1090 Vienna, Austria; 3 Department of Neurosurgery, University of Vienna, A-1090 Vienna, Austria; 4 Department of Radiooncology, University of Vienna, A-1090 Vienna, Austria; 5 Clinical Institute for Neurology, University of Vienna, A-1090 Vienna, Austria

**Keywords:** glioma, chemotherapy, dacarbazine, fotemustine, glioblastoma multiforme, long-term survival

## Abstract

A total of 55 patients with histologically proven glioblastoma multiforme (total gross resection: *n*=24, subtotal resection: *n*=20, stereotactic biopsy: *n*=11) were treated with the combination of dacarbazine (D) (200 mg m^−2^) and fotemustine (F) (100 mg m^−2^) and concomitant radiotherapy (2 Gy day^−1^, 5 days per week using limited fields up to 60 Gy) to assess efficacy and toxicity of this regimen. Survival (median survival, 12-, 18- and 24-month survival rates) and time to progression (median time to progression (TTP), 6-month progression-free survival) were analysed by Kaplan–Meier's method. A total of 268 (range 1–8, median: 5) cycles were administered. Median survival is 14.5+ (range: 0.5–40+) months, and the 12-, 18- and 24-month survival rates are 58, 29 and 23%, respectively. Median TTP from the start of D/F therapy is 9.5+ (range: 0.5–33+) months. The 6-month progression-free survival is 54%. Partial remissions were observed in 3.6%. Main toxicity was thrombocytopenia. Five patients were excluded from further D/F application, four patients because of prolonged thrombocytopenia NCI-CTC grades 3 and 4 and one patient because of whole body erythrodermia. One patient died because of septic fever during thrombocytopenia and leukopenia NCI-CTC grade 4 after the first cycle. No other toxicities of NCI-CTC grade 3 or 4 occurred. The treatment is feasible in a complete outpatient setting and the results of the D/F regimen justify further investigations with these compounds.

Generally, the clinical course of patients with newly diagnosed glioblastoma multiforme (GBM) is rapid and fatal with a median survival of less than 1 year.

Standard therapy consists of surgical tumour reduction when feasible, followed by radiotherapy up to a total dose of 60 Gy. Both strategies are associated with a significant survival benefit (Walker *et al*, 1980; Simpson *et al*, 1993; Hess, 1999). Although a significant increase in survival has been noted in two meta-analysis (Fine *et al*, 1993; Glioma Meta-analysis Trialists Group, 2002), the effect of chemotherapy onto survival and progression has been discussed controversially until the recent presentation of the meta-analysis of the Glioma Meta-analysis Trialists Group (Glioma Meta-analysis Trialists (GMT) Group, 2002). Therein, a significant increase in 1-year survival from 40 to 46% is demonstrated. In addition, chemotherapy consistently increases the proportion of long-term survivors from less than 5% approximately 15–20% (Walker *et al*, 1980; Chang *et al*, 1983; Green *et al*, 1983; Fine *et al*, 1993; Salcman *et al*, 1994; De Angelis *et al*, 1998).

The chemotherapeutic agent best studied in GBM has been carmustine (Levin *et al*, 1990; Prados, 2000). To date, no other drug has been proved to be superior. In comparison with carmustine, the combination of procarbazine/lomustine/vincristine failed to increase survival but resulted in more acute toxicity (Prados *et al*, 1999).

However, improved treatment paradigms for GBM are continually being sought. Currently, the substance investigated most eagerly is temozolomide. An ongoing international, randomised trial will reveal whether temozolomide produces a significant survival advantage in newly diagnosed GBM (Stupp and Newlands, 2001), after it has shown promise in several adjuvant trials (Newlands *et al*, 1992,^1996^; Friedman *et al*, 1998; Stupp *et al*, 2000).

In our present study, we concentrated on a more traditional imidazotetrazine derivate, dacarbazine (D), in combination with fotemustine (F), based on the following rationale.

D is a well-tolerated imidazotetrazine derivate (a synthetic analogue of the naturally occurring purine precursor E-amino-1H-imidazole-4-carboxamide) with proven efficacy in recurrent gliomas (Mahaley, 1991). Furthermore, a synergistic effect among the combination of D and F was observed in melanoma cell lines, in patients with disseminated malignant melanoma and recurrent GBM (Fischel *et al*, 1990; Aamdal *et al*, 1992; Avril *et al*, 1990). F, (diethyl 1-(3-(2 chloroethyl) 3 nitrosoureido) ethyl phosphonate) is an alkylating agent characterised by the grafting of a phosphonoalanine group onto the nitrosourea radical with consequent high lipophilia and a high brain permeability coefficient. The improved diffusion through the cell membrane and the blood–brain barrier implies favourable tissue distribution on cerebral tumour lesions (Kayat *et al*, 1987). F yielded a 26% response rate in recurrent malignant gliomas (Frenay *et al*, 1991) and response rates between 25 and 28% in patients with cerebral metastases of malignant melanoma (Jacquillat *et al*, 1990a,1990b). In patients with unresectable GBM, a response rate of 27% could be achieved by combining F with cisplatin and etoposid (Frenay *et al*, 2000). An own second-line study provides data of the D/F combination in recurrent, nitrosourea-pretreated and previously irradiated GBM, yielding a median survival of 45+ (range: 11–142+) weeks (Fazeny-Dörner *et al*, 2000).

These encouraging results prompted us to investigate the combination of dacarbazine and fotemustine (D/F) with concomitant radiotherapy into first-line treatment with the aim to assess efficacy and toxicity in newly diagnosed GBM.

## PATIENTS AND METHODS

### Eligibility criteria

Patients with newly diagnosed, histologically proven GBM, based on the WHO classification (Kleihues and Sobin, 2000) after total gross resection, subtotal resection or after stereotactic biopsy, were eligible for inclusion into this study. For the evaluation of any residual disease a cranial computed tomographic (CT) scan or magnetic resonance imaging (MRI) had to be performed within 72 h after neurosurgical procedure. Patients had to be aged between 18 and 70 years, had to have a Karnofsky performance score (KPS) ⩾60% and a life expectancy of >8 weeks; patients were not allowed to be under cytotoxic chemotherapy because of concurrent malignancy. Other contraindications included any known psychiatric disorder and pregnant or nursing women. Adequate contraception was mandatory.

Radio- and chemotherapy had to be started within 10–14 days after neurosurgical intervention in case of controlled wound healing and missing signs of recent infection (white blood cell counts, C-reactive protein and fibrinogen had to be within normal range according to institutional standard).

Patients had to be on a stable dose of glucocorticoids (or no glucocorticoids) for at least 1 week prior to study entry. Furthermore, patients were required to have adequate liver function (SGOT, SGPT and alkaline phosphatase levels less than two times of institutional normal and bilirubin levels <1.5 mg dl^−1^), renal (blood urea nitrogen or creatinine levels <1.5 times of institutional normal) and bone marrow function (leukocyte count >3000 *μ*l^–1^ and a platelet count >100 000 *μ*l^−1^) before start of D/F therapy. All patients provided written informed consent before study entry.

### Study endpoints

The study end points were efficacy of the D/F regimen concomitant to radiotherapy defined as response to chemotherapy, time to progression (TTP), 6-month progression-free survival, median survival, and 12-, 18- and 24-month survival rates.

### Therapeutic protocol

Chemotherapy consisted of D in a dosage of 200 mg m^−2^ (diluted in 250 ml normal saline) and F in a dosage of 100 mg m^−2^ (diluted in 250 ml glucose 5%). Both solutions were protected from light and were given intravenously in an outpatient setting. D was administered over 30 min, to avoid burning sensations during D infusion, and 500 ml normal saline was concomitantly administered. At 30 min after termination of D infusion, F was given for over 60 min. Cycles were repeated every 3 weeks. Treatment was continued for a maximum of eight cycles, unless there was progression of disease, unmanageable toxicity, fulfilled off-study criteria or withdrawal of consent.

Modification of the doses or the dose interval of D/F was made for haematologic toxicity based on the platelet and leukocyte count on the day of the planned treatment. D/F treatment was postponed up to a maximum of three consecutive weeks in case of thrombocytopenia from the National Cancer Institue (NCI) common toxicity criteria (CTC) (NCI, 1988) grade 1 and/or leukopenia NCI-CTC grade 2 with weekly monitoring of blood cell counts; otherwise the doses of D/F were decreased by 25% each. Patients were excluded from further D/F treatment in the case of thrombocytopenia NCI-CTC grade 2 lasting longer than 3 weeks or immediately after thrombocytopenia or leukopenia NCI-CTC grade ⩾3. After exclusion from study patients were allowed to be treated individually with previously unemployed drugs. Repeat surgery was not considered routinely.

Antiemetics were administered to all patients before and after chemotherapy application according to the institutional standard (granisetron 5 mg orally once a day from days 1–3 after D/F therapy).

Doses of glucocorticoids (dexamethasone) were adjusted according to the patients' clinical status and were given in the lowest dose necessary for neurologic stability. If the dosage was increased to offset marked clinical deterioration, this was considered when evaluating response, using the criteria of MacDonald *et al*. (1990). Concomitantly ranititidin 300 mg orally was given once daily. Anticonvulsants were used as medically indicated.

### Toxicity evaluation

Toxicity was evaluated according to the NCI's common CTC (NCI, 1988) during routine controls in three-weekly intervals or, if clinically indicated, in weekly intervals.

Monitoring of serum chemistry and blood cell counts was performed prior to each cycle of therapy in three-weekly intervals. In case of haematotoxicity necessitating a delay of chemotherapy application, blood counts were performed in weekly intervals.

Patients were monitored with either cranial CT or MRI scan after two, four, six and eight cycles of therapy in case of clinical and neurological stability, and immediately when disease progression was suspected clinically.

### Response evaluation

Response evaluation was based on MacDonald's criteria (MacDonald *et al*, 1990): complete response (CR) was defined as the disappearance of all measurable disease with improved neurology in the absence of corticoid therapy. Partial response (PR) was a ⩾50% decrease in tumour size with an improved or stable neurology on stable or decreased dexamethasone dose. Stable disease (SD) was a less than 50% decrease, or less than 25% increase of the tumour size with an improved or stable neurology on stable or decreased dexamethasone dose. Progressive disease (PD) was a greater than 25% increase in tumour size or the appearance of new lesions. Tumour evaluation was based on the product of the two largest perpendicular diameters of the contrasting lesion. If the tumour did not enhance, the diameters of the hyperintense signal on T-2-weighted MRI images or of the hypodense region on CT scans were used.

### Off-study criteria

Patients were excluded from further D/F treatment if one of the following was noted: (1) disease progression or recurrence as documented by CT or MRI anytime after the completion of at least one cycle of therapy as defined above; (2) severe and/or prolonged haematotoxicity as defined above; (3) in case of a deteriorated and unacceptable neurologic status; (4) in case of withdrawal of consent.

### Statistical analysis

All analyses were done by intention to treat. The reference point for median survival, 12-, 18- and 24-month survival, was the date of neuropathologic diagnosis, and the end point was survival until death, including deaths from causes not related to the disease.

Time to progression (TTP) was estimated from the first day of D/F application to the first unfavourable event (e.g. radiographically documented tumour recurrence or progression or death). If a patient died without a scan to document disease status, the TTP was measured until documented clinical worsening or until the date of death.

Survival curves and TTP curves were constructed using the Kaplan–Meier's nonparametric method, medians (and their respective 95% confidence intervals) were calculated from the Kaplan–Meier estimates (Kaplan and Meier, 1958; Young *et al*, 1999). Statistical evaluations were performed with SPSS version 10.0.7 program package. Data were analysed as of 31 January 2002.

## RESULTS

A total of 55 patients (female/male: 16/39) with newly diagnosed, histologically proven GBM were treated with D/F chemotherapy between October 1998 and December 2001. In total, 24 patients had gross total resection, 20 patients had a subtotal tumour resection and in 11 patients stereotactic biopsy was performed. Concomitant to chemotherapy all patients received radiotherapy up to a total dose of 60 Gy (30×2 Gy single dose).

Median age was 44 (range: 18–68) years, the median KPS 1 week after neurosurgical intervention was 90% (range: 60–100%).

### Survival

**Figure 1:**
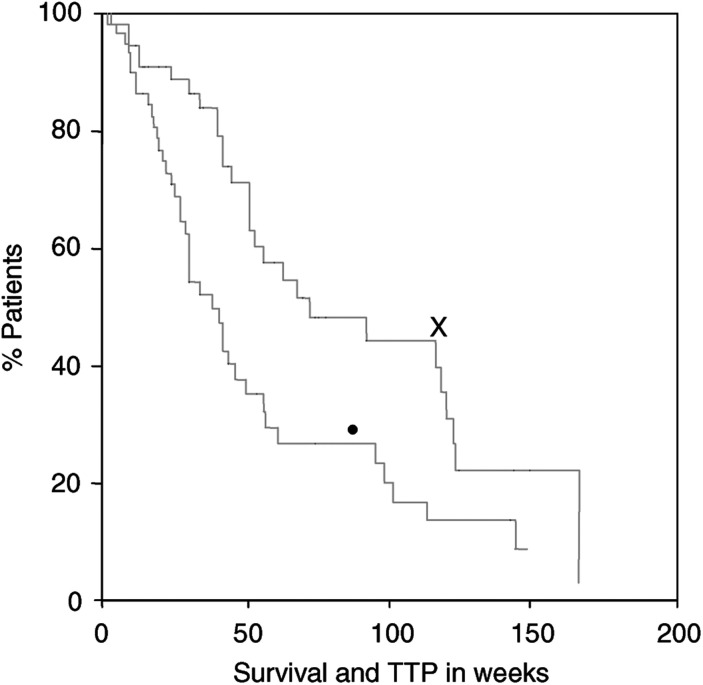
Survival and TTP of patients with GBM after first-line therapy with D and F: **×**, survival; K, •, time to progression (TTP).

At study evaluation 23 out of 55 (42%) patients are alive. Median survival of all patients (Figure 1

) is 72+ (range: 3–156) weeks (=14.5 months in median) with a 95% confidence interval of 28–116 weeks. The 12-, 18- and 24-month survival rates after diagnosis are 58, 29 and 23%, respectively.

### Time to progression

The median TTP of all patients was 41+ (range: 3–144+) weeks (=9.5 months) (Figure 1).

### Response to chemotherapy

A partial remission could be achieved in two patients (3.6%) with subtotal resection, lasting for 12 weeks each. SD or no evidence of disease lasting from 10 to 144+ weeks (median: 29 weeks) could be achieved in 45 (81%) of the patients. Thereof, 24 patients had gross total resection, 14 patients had subtotal resection and seven patients had stereotactic biopsy.

SD or no evidence of disease lasting longer than 12 months was observed in 12 (22%) patients. In these patients, glucocorticoids could be tapered towards the end of radiotherapy and usually stopped within 3 weeks after radiotherapy. Additionally, in these patients the KPS improved at least by 10%.

### Toxicity

In all, 55 patients received a total of 268 (range 1–8; median: 5) cycles of D/F.

Major toxicity was thrombocytopenia. Four patients were excluded from further D/F treatment, three because of thrombocytopenia NCI-CTC grade 3 (after the second, fourth and sixth cycle, respectively) and the other patient because of thrombo-cytopenia of NCI-CTC grade 4 (after the eigth cycle of D/F), which was accompanied by anaemia NCI-CTC grade 3, necessitating substitution of platelets and two units of packed red cells.

One patient died owing to septic fever during combined thrombocytopenia and leukopenia NCI-CTC grade 4 after the first D/F application despite inward care, intravenous administration of antibiotics, granulocyte colony-stimulating factors and substitution of platelets. Cranial CT before death showed stable disease of the unresectable tumour.

Thrombocytopenia NCI-CTC grade 1 was observed in 11 cycles (therapy was postponed for 1 week in seven cycles and for 2 weeks in four cycles) and thrombocytopenia NCI-CTC grade 2 occurred in 13 cycles (therapy was postponed for 1 week in five cycles, for 2 weeks in five cycles, and for 3 weeks in three cycles resulting in a 25% dose reduction of D/F). Leukopenia from NCI-CTC grade 1 occurred in six cycles (three cycles had to be postponed for 1 week) and from CTC grade 2 in six cycles, resulting in treatment delay for 1 week in three cycles, for 2 weeks in one cycle and in a 3 weeks delay in two cycles, thus leading to a 25% dose reduction of D/F.

A fifth patient was excluded from further therapy because of a whole body erythordermia within 24 h after administration of the second cycle of D/F.

Alopecia was evaluable in 55 patients. The most severe degree was NCI-CTC grade 2 (*n*=18).

Neither pulmonary toxicity nor any radiation necrosis was observed during a follow-up period of 150 weeks. Under standardised prophylactic antiemetics, patients did not suffer from gastrointestinal toxicity. The main complaints of patients concerned side effects from chronic glucocorticoid intake, primarily the cushingoid appearance, myopathy and the vulnerability of the skin.

## DISCUSSION

The adjuvant administration of D/F concomitant to radiotherapy revealed to be feasible in a complete outpatient setting and yielded a median survival of 14.5 months in patients with newly diagnosed GBM. Our survival and TTP results range in the upper level of those achieved in the limited number of available first-line treatment strategies, which are summarized in Table 1

**Table 1:** Synopsis of first-line chemotherapy trials in patients with newly diagnosed GBM

**Author**	**No. of patients with GBM out of all patients**	**Agent and treatment design**	**Median TTP (months)**	**Median survival (months)**	**Remarks**
Beauchesne *et al* (1999)	23/30	(Etoposide 100 mg m^−2^ d1–3) q 4 weeks; ×6	7	12	TTP + survival analysis for all 30 patients ‘near-concurrent’ radiotherapy
				18-month survival: 15.4%	
				24-month survival: 4%	
Brandes *et al* (1998)	56/56	(Carboplatin 350 mg m^−2^ d1,22,43+	7.5	12.5	Concurrent radiotherapy (60 Gy)
		Teniposide 50 mg m^−2^ d1–3,22–24,43–45 +		18-month survival: 28%	
		Carmustine 200 mg m^−2^) q 8 weeks; ×3		24-month survival: 20%	
Fetell *et al* (1997)	33/33	(Paclitaxel 140–230 mg m^−2^ d1) q 21 days; ×3	n.e.	12.5	Radiotherapy at tumour progression
Frenay *et al* (2000)	33/33	(Fotemustine 100 mg m^−2^ d1+	n.e.	10	Symptomatic unresectable GBM
		Cisplatin 33 mg m^−2^ d1–3 +			
		Vepesid 75 mg m^−2^ d1–3) q 28 days			
		q 28 days			
Friedmann *et al* (1998)	33/33	(Temozolomide 200 mg m^−2^ d1–5, p.o.); q 28 days; ×4	7 months in responding patients (*n*=17)	12 months in responding patients	Residual tumour mass after surgery;
			2 months in non-responding patients (*n*=16)	6 months in non-responding patients	Chemotherapy before radiotherapy
Friedman *et al* (1999)	14/63	(Topotecan 2.6 mg m^−2^ over 72 h) q weekly; ×16	n.e.	n.e.	Chemotherapy before radiotherapy
					Partial response in 2/14 patients
Grossman *et al* (2000)	219/219	(1) BCNU+cisplatin *vs*	(1): 5.4	(1): 10.7	No advantage from (1) over (2)
		(2) BCNU+radiotherapy	(2): 4.2	(2): 11.2	
Gruber *et al* (1998)	25/25	(carboplatin 600 mg m^−2^) q 4 weeks; ×4	8.4	19.2	Radiotherapy (60 Gy) started 3–4 weeks after chemotherapy; 23/25 patients: KPS:100%
Stupp *et al* (2000)	45/45	(Temozolomide 75–200 mg m^−2^d×5d) q 28 days	n.e	Estimated 1-year survival: 67%	Concomitant radiotherapy
This paper	55/55	(Dacarbazine 200 mg m^−2^ d1+	9.5+	14.5+	Concomitant radiotherapy (60Gy)
		Fotemustine 100 mg m^−2^ d1)		18-month survival: 29%	
		q 21days;		24-month survival: 23%	61% of patients alive at study evaluation

. Therein, the median TTP is 7 (range: 4.2–9.5) months and the median survival is 12 (range: 9–19.2) months (Table 1; Fetell *et al*, 1997; Brandes *et al*, 1998; Friedman *et al*, 1998; Gruber *et al*, 1998; Beauchesne *et al*, 1999; Frenay *et al*, 2000; Grossman *et al*, 2000; Stupp *et al*, 2000).

In particular, our study, administering the traditional imidazotetrazinederivate, dacarbazine, is comparable with Stupp's preliminary study, administering the imidazotetrazinederivate temozolomide (Stupp *et al*, 2000). Both imidazotertazinederivate studies are similar concerning study design, extent of surgery and the median Karnofsky performance score. Although the measurable partial response of 3.6% is discouraging, the definitive 12-month survival achieved in our study is 58%, whereas the estimated 12-month survival for the preliminary temozolomide study has been reported to be 67%. It might be speculated whether the younger median age of our patients might have contributed to the favourable outcome. However, the recent meta-analysis of the Glioma Meta-analysis Trialists Group (Glioma Meta-analysis Trialists (GMT) Group, 2002) demonstrated that the relative effect of chemotherapy on 1-year survival does not differ between patients ⩾40 years and those ⩾60 years.

The 18-month survival is the usual marker for beneficial influence of adjuvant chemotherapy (De Angelis *et al*, 1998). It is documented in two studies of the synopsis summarised in Table 1 and ranges between 15.4 and 28% and is 29% in our study (Brandes *et al*, 1998; Beauchesne *et al*, 1999). Concerning median survival, Gruber *et al* (1998) achieved 19.2 months with the single agent carboplatinum. However, 18- and 24-month survival rates are not reported in their study and a study populat-ion smaller than 30 patients is known to preclude strong statistical power.

An explanation for the efficacy of the D/F combination might be the *O*6-alkyl synergy of D/F, and the augmentation thereof by the excellent tissue distribution of F in cerebral tumour lesions on the other hand (Jacquillat *et al*, 1990a,1990b; Lee *et al*, 1991; Aamdal *et al*, 1992; Frenay *et al*, 2000).

One case of fatal neutropenic fever after the first cycle of therapy was unexpected. Neither advanced age nor a dismal pretherapeutic KPS could explain this adverse event (Grant *et al*, 1995; Raymond *et al*, 1996). No other case of serious toxicity than uncomplicated leuco- and/or thrombocytopenia from NCI-CTC grades 3 and 4 (9%) occurred during the application of these 268 D/F cycles as well as in further 100 D/F applications in patients with recurrent and lomustine pretreated GBM (Fazeny-Dörner *et al*, 2000). Otherwise, the toxicity of D/F was similar to the previously reported experience with these compounds, thus making the regimen suitable for completely outward administration (Jacquillat *et al*, 1990a,1990b; Aamdal *et al*, 1992; Fazeny-Dörner *et al*, 2000; Raymond *et al*, 1996).

The results of the D/F regimen justify further investigations with these compounds.
